# Mitochondrial calcium signaling and redox homeostasis in cardiac health and disease

**DOI:** 10.3389/fmmed.2023.1235188

**Published:** 2023-08-23

**Authors:** Tudor-Alexandru Popoiu, Christoph Maack, Edoardo Bertero

**Affiliations:** ^1^ Department of Translational Research, Comprehensive Heart Failure Center, University Clinic Würzburg, Würzburg, Germany; ^2^ “Victor Babes” University of Medicine and Pharmacy, Timisoara, Romania; ^3^ Chair of Cardiovascular Disease, Department of Internal Medicine and Specialties, University of Genoa, Genova, Italy

**Keywords:** mitochondria, cardiomyocyte, calcium, redox homeostasis, heart failure, reactive oxygen species

## Abstract

The energy demand of cardiomyocytes changes continuously in response to variations in cardiac workload. Cardiac excitation-contraction coupling is fueled primarily by adenosine triphosphate (ATP) production by oxidative phosphorylation in mitochondria. The rate of mitochondrial oxidative metabolism is matched to the rate of ATP consumption in the cytosol by the parallel activation of oxidative phosphorylation by calcium (Ca^2+^) and adenosine diphosphate (ADP). During cardiac workload transitions, Ca^2+^ accumulates in the mitochondrial matrix, where it stimulates the activity of the tricarboxylic acid cycle. In this review, we describe how mitochondria internalize and extrude Ca^2+^, the relevance of this process for ATP production and redox homeostasis in the healthy heart, and how derangements in ion handling cause mitochondrial and cardiomyocyte dysfunction in heart failure.

## 1 Introduction

Calcium (Ca^2+^) signaling plays a central role in the contractile, metabolic, and epigenetic functions of cardiac myocytes. Transient variations in cytosolic Ca^2+^ concentration ([Ca^2+^]_c_) trigger cardiac myocyte contraction and relaxation. During physiological elevations of cardiac workload, the accumulation of Ca^2+^ in the mitochondrial matrix adjusts the rate of mitochondrial oxidative metabolism to the energy requirements of the cell (McCormack and Denton, 1993). In cardiac disease, Ca^2+^ mishandling can wreak havoc on cellular functions: in heart failure (HF), defective mitochondrial Ca^2+^ accumulation can cause bioenergetic mismatch and oxidative stress (Kohlhaas et al., 2017); in ischemia/reperfusion (I/R) injury, mitochondrial Ca^2+^ overload leads to a sudden increase in mitochondrial membrane permeability that is one major driver of cell death (Xuxia et al., 2020). In addition to cardiac myocyte contraction, changes in [Ca^2+^]_c_ modulate the activation of gene expression, a process coined *excitation-transcription coupling*. Here, we review the role of Ca^2+^ signaling as a regulator of mitochondrial oxidative metabolism and reactive oxygen species (ROS) emission from mitochondria, and how derangements in these processes contribute to the development of HF.

## 2 Cardiac mechano-energetic coupling

In cardiac myocytes, Ca^2+^ handling is mediated by voltage- and ligand-activated ion channels that are mainly found on the sarcolemma and on the sarcoplasmic reticulum (SR), a specialized endoplasmic reticulum that cyclically releases and removes Ca^2+^ ions from the cytosol during cardiac myocyte contraction and relaxation. During phase 2 of the action potential, opening of voltage-dependent L-type Ca^2+^ channels on the sarcolemma leads to Ca^2+^ entry into the cytosol, which in turn triggers the release of a larger amount of Ca^2+^ from the SR via type 2 ryanodine receptors (RyR2). This phenomenon, termed *Ca* ^*2+*^ *-induced Ca* ^*2+*^ *release,* initiates contraction as Ca^2+^ binds the regulatory protein troponin C, thereby inducing a conformational change that enables the interaction of the myosin head with actin (Bers, 2002). During diastole, Ca^2+^ is removed from the cytosol by the SR Ca^2+^ ATPase (SERCA), which takes Ca^2+^ back into the SR, and to a lesser extent by the sarcolemmal sodium (Na^+^)/Ca^2+^ exchanger (NCX), which extrudes Ca^2+^ to the extracellular space; for each Ca^2+^ ion removed, the NCX imports 3 Na^+^ ions in the cytosol.

**FIGURE 1:**
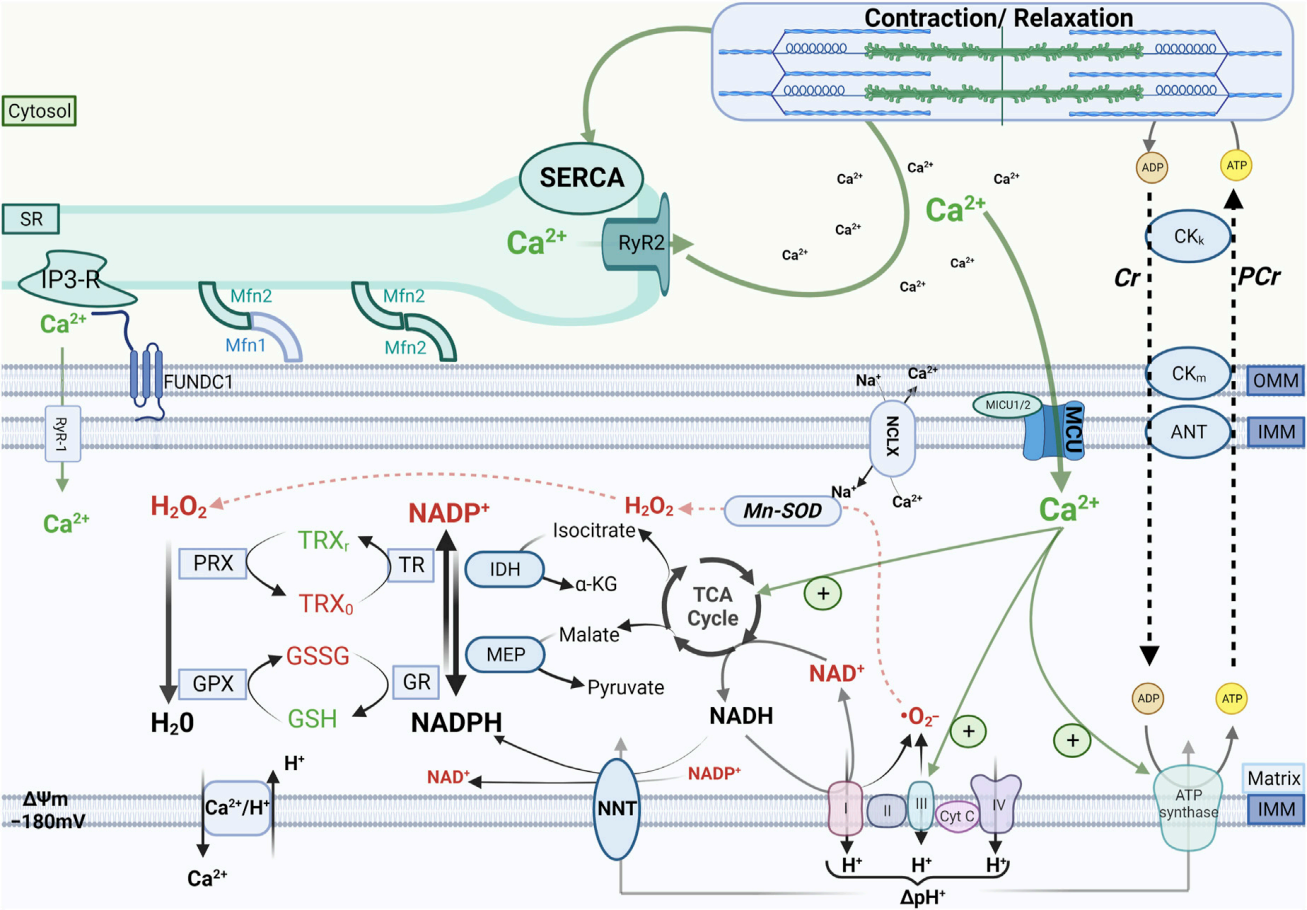
Cardiac mechano-energetic coupling. In the healthy heart, the tricarboxylic acid (TCA) cycle produces the reduced form of nicotinamide adenine dinucleotide (NADH) and nicotinamide adenine dinucleotide phosphate (NADPH). Electrons donated by NADH at complex I or by succinate at complex II of the respiratory chain are channeled through a series of electron acceptors that harness their energy to pump protons from the matrix to the intermembrane space. This process physiologically produces a certain amount of reactive oxygen species (ROS) mainly due to incomplete reduction of oxygen to superoxide (^.^O_2_^−^), which is rapidly converted to hydrogen peroxide (H_2_O_2_) by the manganese-dependent superoxide dismutase (Mn-SOD). In turn, H_2_O_2_ is reduced to H_2_O by peroxiredoxin (PRX) and glutathione peroxidase (GPX), which are regenerated in their active (reduced) form by a cascade of redox reactions fueled by NADPH. The main sources of mitochondrial NADPH are the TCA cycle enzymes isocitrate dehydrogenase and malate dehydrogenase and the nicotinamide nucleotide transhydrogenase (NNT). When adenosine triphosphate (ATP) consumption increases, increased flux of adenosine diphosphate (ADP) to mitochondria via the creatine kinase (CK) shuttle and Ca^2+^ accumulation in the mitochondrial matrix stimulate oxidative phosphorylation and the TCA cycle activity, respectively, thereby matching ATP supply and demand and providing reducing equivalents to maintain the redox state of mitochondrial antioxidant systems. Other abbreviations: SR, sarcoplasmic reticulum; RyR1 and RyR2, ryanodine receptors type 1 and type 2, respectively; MCU, mitochondrial calcium uniporter; NCLX, Na^+^/Ca^2+^ exchanger, IP3-R, inositol 1,4,5-trisphosphate receptors; Mfn1 and Mfn2, mitofusin 1 and mitofusin 2, respectively; IMM and OMM, inner and outer mitochondrial membrane, respectively; FUNDC1, FUN14 domain containing 1; Cr, creatine; PCr, phosphocreatine; TRX, thioredoxin; GSH and GSSG, reduced and oxidized glutathione, respectively; GR, glutathione reductase; TR, thioredoxin reductase; IDH, isocitrate dehydrogenase, MEP, malic enzyme; NNT, nicotinamide nucleotide transhydrogenase; SERCA, SR Ca^2+^ ATPase.

Ca^2+^ operates as a second messenger to adjust the rate of mitochondrial oxidative metabolism according to the energy requirements of cardiac myocytes (McCormack and Denton, 1993; Brandes and Bers, 1997). Increases in heart rate and contractility as seen during physical exercise increase the rate of adenosine triphosphate (ATP) turnover by cardiac myocytes up to 5- or 6-fold the baseline rate. The increase in adenosine diphosphate delivery to mitochondria via the creatine kinase system accelerates the rate of ATP production by oxidative phosphorylation, which needs to be sustained by an increased supply of reducing equivalents that are donated to complex I and complex II of the electron transport chain (ETC) by the reduced form of nicotinamide adenine dinucleotide (NADH) and succinate, respectively, derived from the tricarboxylic acid (TCA) cycle. Ca^2+^ is pivotal to this *mechano-energetic coupling* by stimulating the TCA cycle dehydrogenases (Figure 1).

Furthermore, elevations in [Ca^2+^]_c_ promote the shuttling of reducing equivalents from cytosolic NADH to the mitochondrial NADH pool by stimulating the activity of the two aspartate/glutamate carriers (AGC1/2, also known as SLC25A12 and SLC25A13 or aralar1 and citrin, respectively) that sense Ca^2+^ levels on the outer face of the IMM via their EF-hand motifs (Palmieri et al., 2001). The efflux of aspartate from the matrix in exchange for glutamate catalyzed by AGC1/2 is one of the steps of the malate-aspartate shuttle, a shuttle system whose net effect is that NADH in the cytosol is oxidized to NAD^+^, and NAD^+^ in the matrix is reduced to NADH. AGC1 is the predominant isoform found in cardiac mitochondria, and it responds to Ca^2+^ concentrations lower than those driving Ca^2+^ accumulation in the mitochondrial matrix. On these grounds, it has been proposed that the Ca^2+^-dependent activation of AGC1 might regenerate NADH to sustain oxidative phosphorylation before matrix Ca^2+^ levels rise (Contreras et al., 2007).

The reducing equivalents derived from oxidation of substrates via the TCA cycle are also used to sustain mitochondrial antioxidant systems. In fact, the reduced form of nicotinamide adenine dinucleotide phosphate (NADPH) serves as an electron donor for the redox reactions that convert hydrogen peroxide (H_2_O_2_) into water. Mitochondrial oxidative metabolism normally produces a certain quantity of ROS, which also serve physiological functions as signaling molecules, but can damage cellular components when produced in exaggerate amounts or not adequately scavenged. The best-characterized sites of ROS formation in mitochondria are complexes I and III of the ETC, where the superoxide radical (^•^O_2_^−^) can be generated by the incomplete reduction of O_2_ (Murphy, 2009). Superoxide is rapidly converted to H_2_O_2_ by the manganese-dependent superoxide dismutase (Mn-SOD). Another source of mitochondrial ROS is monoamine oxidases, a family of flavoprotein residing in the outer mitochondrial membrane that degrades endogenous monoamines using flavin adenine dinucleotide (FAD) as a cofactor and generating H_2_O_2_ as a product (Kaludercic et al., 2011). In turn, H_2_O_2_ is reduced to water by glutathione peroxidase or peroxiredoxin, which then need to be regenerated in their active (reduced) form by a cascade of redox reactions that are fueled by NADPH. Because the majority of mitochondrial NADPH is produced by reactions that use TCA cycle intermediates as substrates, the Ca^2+^-dependent stimulation of the TCA cycle is pivotal to maintain an adequate supply of reducing equivalents to both the ETC and H_2_O_2_-eliminating systems during elevations of cardiac workload (Figure 1).

Efficient Ca^2+^ uptake by mitochondria is made possible by the spatial proximity between mitochondria and the SR. Contact points between mitochondria and the SR create spatially localized microdomains where micromolar Ca^2+^ concentrations are transiently attained after Ca^2+^ is released from the SR. These local elevations of Ca^2+^ concentration are essential for efficient mitochondrial Ca^2+^ uptake (Giacomello et al., 2010). The regions of the SR that are tethered to mitochondria are also known as mitochondria-associated membranes (MAM), and the integrity of these contact sites is maintained by specialized proteins such as mitofusin 2 (Mfn2), a dynamin-related GTPase that is expressed on both the outer mitochondrial and SR membranes. Loss of Mfn2-mediated interorganelle tethering in the heart reduces the extent of mitochondria-SR contact sites, thereby impairing mitochondrial Ca^2+^ uptake and the Ca^2+^-dependent metabolic adaptation in response to β-adrenergic stimulation (Chen et al., 2012). Viceversa, chronic enhancement of cardiac mitochondria-SR tethering via expression of a cardiac myocyte-specific tether transgene potentiates mitochondrial-SR Ca^2+^ crosstalk, decreasing cardiac vulnerability to adrenergic stress and I/R injury (Nichtová et al., 2023).

Other proteins, such as PACS-2 (phosphofurin acidic cluster sorting protein 2), sigma-1 receptor, GRP75 (glucose-regulated protein 75), and FUNDC1 (FUN14 domain containing 1) were also identified as potential mitochondria-SR tethering proteins and shown to play a role in the communication between SR and mitochondria. Genetic deletion of these proteins results in cardiac dysfunction, ER stress, and mitochondrial fragmentation (Szabadkai et al., 2006; Hayashi and Su, 2007; Stoica et al., 2014; Wu et al., 2017). The ability of the SR to store large amounts of Ca^2+^ ions depends on the presence of Ca^2+^-binding proteins such as calsequestrin (CASQ) in its lumen. CASQ acts as a Ca^2+^ buffer that maintains the SR luminal free Ca^2+^ concentration between 100–500 μM. The expression of CASQ can be enhanced or suppressed at a gene level, resulting in an increase or decrease in SR Ca^2+^ load. In addition to acting as a major Ca^2+^ storage protein, CASQ also regulates the activity of the RyR2 channels (Fearnley et al., 2011).

Overall, there is a tight interplay between the ATP-consuming processes of excitation-contraction coupling and the ATP-producing processes of mitochondrial oxidative metabolism. Ca^2+^ plays a central role in matching cardiac energy supply to demand and sustaining mitochondrial antioxidative capacity during transitions of cardiac workload.

## 3 Mitochondrial Ca^2+^ handling

### 3.1 The mitochondrial Ca^2+^ uniporter complex

Mitochondrial Ca^2+^ uptake is mediated by a macromolecular complex embedded in the inner mitochondrial membrane (IMM) and coined the mitochondrial Ca^2+^ uniporter (MCU) complex. The MCU pore consists of a tetramer of 35-kDa MCUa subunits, which enclose a conserved DIME motif that accounts for the Ca^2+^ selectivity of the channel (Yoo et al., 2018; De Stefani et al., 2011). MCUa has a dominant negative homolog, coined MCUb, characterized by differences in the DIME motif that disrupt the Ca^2+^ permeability of the pore (Raffaello et al., 2013). The key difference between MCUa and MCUb is the replacement of Glu257 with Val242 in MCUb, which results in the loss of a negative charge and thus hinders the electrostatic attraction for Ca^2+^ (Colussi and Stathopulos, 2022). Therefore, MCUb operates as a negative regulator of mitochondrial Ca^2+^ uptake. Under physiological conditions, the MCU complex of cardiac myocytes does not comprise MCUb subunits, but these are incorporated in the MCU under pathological conditions, such as ischemic injury (Lambert et al., 2019). By reducing mitochondrial Ca^2+^ influx, MCUb renders mitochondria less sensitive to Ca^2+^ overload and consequent permeability transition. Indeed, MCUb overexpression reduces infarct size after I/R injury in mice (Lambert et al., 2019), and its upregulation after myocardial infarction might represent a cardioprotective mechanism to attenuate post-ischemic ventricular remodeling (Huo et al., 2020).

To protect mitochondria from the catastrophic effects of Ca^2+^ overload, MCU conductance is fine-tuned by several regulatory subunits, such as the EF-hand-containing proteins of the MICU family. MICU1 forms heterodimers with either MICU2 or MICU3 and modulates MCU flux depending on Ca^2+^ concentration in the intermembrane space (Wu et al., 2020). Although the mechanisms underlying the MICU-mediated regulation of MCU flux have not been completely elucidated, the prevailing view is that MICU1 occludes the cytoplasmic entry of the MCU pore at submicromolar [Ca^2+^]_c_, and moves away from the pore to permit Ca^2+^ penetration when [Ca^2+^]_c_ increase to the micromolar range (Van Keuren et al., 2020). Therefore, the MICU proteins function as gatekeepers to prevent mitochondrial Ca^2+^ overload (Mallilankaraman et al., 2012).

The essential MCU regulatory element (EMRE) is a core component of the MCU complex. EMRE induces conformational changes in the pore domain that allow Ca^2+^ transit through the channel (Sancak et al., 2013). It was initially thought that the stoichiometry of MCUa and EMRE is in 1:1 ratio, but recent studies suggest that there are fewer EMRE subunits than MCUa subunits, with significant variability between different tissues (Watanabe et al., 2022). Upon EMRE binding to the MCU, the MCU-EMRE complex forms a V-shaped dimer, changing the structural organization of the MCU complex and allowing Ca^2+^ influx through the channel pore (Wang et al., 2019). EMRE downregulation might represent an endogenous mechanism to limit mitochondrial Ca^2+^ overload under conditions that chronically elevate MCU flux, such as in the MICU1-knockout mouse model (Liu et al., 2016).

MCU regulator 1 (MCUR1) was proposed as another putative component of the uniporter complex, but evidence in this regard is controversial: although MCUR1 modulates Ca^2+^ threshold for mitochondrial permeability transition and its deletion alters Ca^2+^ uptake and MCU current (Chaudhuri et al., 2016; Tomar et al., 2016), it was proposed that MCUR1 is in fact a cytochrome *c* oxidase (complex IV) assembly factor and not a regulator of the MCU (Paupe et al., 2015). Furthermore, a role in the regulation of the MCU current has been proposed for the solute carrier family 25 member 3 (SLC25A3), a metabolite transporter that interacts with MICU1 and MCU (Hoffman et al., 2014; Alevriadou et al., 2021).

The MCU complex has a low conductance and high Ca^2+^ selectivity. The complex is found in most tissues, but there are major tissue-specific differences in MCU fluxes that reflect the variability in current density and channel stoichiometry. In particular, the MICU1/MCU ratio is a key determinant of tissue-specific differences in the [Ca^2+^]_c_ threshold for Ca^2+^ uptake and activation of oxidative metabolism (Paillard et al., 2017). MCU current density is the highest in skeletal muscle mitochondria and the lowest in cardiac mitochondria (Fieni et al., 2012; Boyman et al., 2021). Skeletal muscle mitochondria display the highest MCU current density, which is 30-fold higher than in cardiac mitochondria. This difference reflects the substantially lower volume occupied by mitochondria in cardiac vs. skeletal myocytes (37% vs. 5%). Moreover, the MCU current density varies also between different ages as it is lower in newborn that in adult mice (Fieni et al., 2012).

### 3.2 The mitochondrial Na^+^/Ca^2+^ exchanger (NCLX)

The main pathway for mitochondrial Ca^2+^ efflux is the mitochondrial Na^+^/Ca^2+^ exchanger, or NCLX (Palty et al., 2010). The existence of a Na^+^/Ca^2+^ exchanger as a primary Ca^2+^ extrusion mechanism from mitochondria implies that mitochondrial Ca^2+^ levels are controlled by cytosolic Na^+^, whereby elevations of intracellular Na^+^ concentration ([Na^+^]_i_) as seen in HF can hinder mitochondrial Ca^2+^ accumulation. Another relevant property of the NCLX is that its kinetics of Ca^2+^ extrusion are slower than Ca^2+^ uptake via the MCU, accounting for the accumulation of Ca^2+^ in the mitochondrial matrix during β-adrenergic stimulation.

The importance of NCLX in regulating mitochondrial Ca^2+^ levels is underscored by studies of genetic manipulation of NCLX expression. On the one hand, cardiac germline deletion of NCLX is embryonically lethal, and when the gene is silenced in adult mice, these develop severe cardiac dysfunction and sudden cardiac death secondary to mitochondrial Ca^2+^ overload (Luongo et al., 2017). Conversely, NCLX overexpression in cardiac myocytes is protective against cardiac maladaptive remodeling and prevents contractile disfunction in mice subjected to pressure overload by means of transverse aortic constriction (TAC) for 12 weeks (Garbincius et al., 2022).

### 3.3 Physiological role of mitochondrial Ca^2+^ uptake in the heart

The Ca^2+^-dependent stimulation of mitochondrial oxidative metabolism enables the heart to adjust cardiac inotropy to the circulatory demand, which is determined by neurohormonal activity. The physiological relevance of MCU-dependent mitochondrial Ca^2+^ uptake was initially investigated in studies using ruthenium derivatives to inhibit the MCU current. In a series of landmark studies conducted in rat cardiac trabeculae, Brandes and Bers demonstrated that elevations of cardiac workload simulated by simultaneous β-adrenergic stimulation and transient increases in pacing frequency transiently oxidize the redox state of the mitochondrial pyridine nucleotides NADH and FADH_2_ (Brandes and Bers, 1996; Brandes and Bers, 1997; Brandes and Bers, 1999; Brandes and Bers, 2002). The subsequent increase in mitochondrial Ca^2+^ restores the redox state of NADH and FADH_2_, but this is substantially blunted or abrogated by blocking the MCU current with the ruthenium derivative Ru360 or by elevating [Na^+^]_i_, which accelerates mitochondrial Ca^2+^ efflux via the NCLX (Maack et al., 2006; Liu and O’Rourke, 2008).

A long-standing debate is whether mitochondrial Ca^2+^ uptake happens on a beat-to-beat basis or more slowly, as a progressive accumulation over several cytosolic Ca^2+^ transients. In the beat-to-beat model, fluctuations in mitochondrial Ca^2+^ levels parallel changes in cytosolic Ca^2+^, and the increase in diastolic Ca^2+^ levels in the mitochondrial matrix induced by β-adrenergic stimulation stimulates the TCA cycle dehydrogenases (O’Rourke and Blatter, 2009; la Fuente and Sheu, 2019). Conversely, other studies argued against the existence of mitochondrial Ca^2+^ transients, suggesting that mitochondria accumulate Ca^2+^ by integrating cytosolic Ca^2+^ signals (Hüser et al., 2000). The controversial results supporting the two models might be explained by differences in experimental conditions, types of Ca^2+^ probes employed, and animal species (Bertero and Maack, 2018).

Following elucidation of the molecular identity of the MCU in the early 2010s, the role of the MCU current in cardiac metabolic adaptation has been further investigated in mouse models of genetic deletion of the MCUa subunit. These studies have shown that MCU deficiency has little or no consequence on cardiac function at rest, but hinders the cardiac chronotropic and inotropic response (Holmström et al., 2015; Kwong et al., 2015; Wu et al., 2015). Furthermore, it was recently discovered that in Barth syndrome, a rare X-linked mitochondrial disorder characterized by abnormal biosynthesis of the mitochondrial phospholipid cardiolipin, the altered composition of the IMM disrupts the structural and functional integrity of several macromolecular complexes embedded in the membrane, including the MCU (Ghosh et al., 2020; Bertero et al., 2021). We studied the functional consequences of MCU loss in a mouse model of Barth syndrome, and discovered that abrogation of mitochondrial Ca^2+^ uptake in cardiac myocytes abolished the cardiac inotropic response to β-adrenergic stimulation (Bertero et al., 2021). Noteworthy, Barth syndrome patients who do not develop overt systolic dysfunction early in life exhibit a blunted cardiac contractile reserve during physical exercise even in the presence near-normal left ventricular ejection fraction (Spencer et al., 2011). Therefore, Barth syndrome might represent the first example of a human disease in which loss of the MCU is a primary pathophysiological mechanism.

By regulating the rate of oxidative metabolism, mitochondrial Ca^2+^ levels induce epigenetic modifications that influence cellular differentiation in certain cell types. In mice, fibroblast-specific MCU deletion exacerbates fibrosis after myocardial infarction or angiotensin II administration (Lombardi et al., 2019). Mechanistically, pro-fibrotic signals such as transforming growth factor (TGF)-β reduce mitochondrial Ca^2+^ uptake by decreasing the MCU current in a MICU1-dependent manner. This leads to an induction of anabolic pathways feeding the TCA cycle that is associated with an increased availability of glutamine-derived α-ketoglutarate (α-KG). In turn, this activates the α-KG-dependent histone demethylases, thereby promoting changes in the structure of chromatin at locations related to the myofibroblast gene program, resulting in cellular differentiation. These findings indicate that the MCU plays a role in regulating the epigenome and influencing cellular differentiation beyond its role in metabolic regulation and cell death (Lombardi et al., 2019).

### 3.4 Alternative pathways of mitochondrial Ca^2+^ uptake and release

The existence and identity of alternative pathways of mitochondrial Ca^2+^ uptake and release is still a matter of debate (Lazaropoulos and Elrod, 2022). Mouse models of MCUa gene silencing showed that mitochondrial Ca^2+^ uptake is not completely abrogated in *Mcua*-knockout mice, although Ca^2+^ uptake kinetics are markedly slower compared with wild-type animals (Kwong et al., 2015; Luongo et al., 2015). It has been suggested that skeletal muscle-type ryanodine receptor type 1 (RyR1) is also found on the IMM of cardiac mitochondria and uptakes Ca^2+^ released from the SR upon stimulation with inositol triphosphate (Seidlmayer et al., 2016). Of note, RyR1 overexpression leads to mitochondrial fragmentation and increased ATP production (O-Uchi et al., 2013).

Furthermore, the existence of one or more Na^+^-independent mechanism(s) of mitochondrial Ca^2+^ efflux has been hypothesized since the 1970s (Carafoli et al., 1974). One recent study demonstrated that the transmembrane BAX inhibitor motif containing protein 5 (TMBIM5) mediates Na^+^-independent Ca^2+^ efflux from the matrix, indicating that it operates as a Ca^2+^/H^+^ exchanger (Austin et al., 2022). TMBIM5 was identified based on its interaction with the leucine zipper EF-hand containing transmembrane protein 1 (LETM1), a single transmembrane protein that was initially characterized as a potassium (K^+^)/H^+^ exchanger (Froschauer et al., 2005) and was subsequently proposed as a Ca^2+^/H^+^ exchanger (Jiang et al., 2009; Shao et al., 2016; Austin and Nowikovsky, 2019). However, its function, ion selectivity (Ca^2+^ vs. K^+^) and mode of operation as a ion transporter remain unclear.

The IMM is also host for the uncoupling proteins 2 and 3 (UCP2 and UCP3) that might operate as Ca^2+^ channels. Depending on the origin and mechanism of the elevation in cytosolic Ca^2+^, UCP2 and UCP3 might also mediate mitochondrial Ca^2+^ uptake. Under physiological conditions, UCP2 and UCP3 facilitate Ca^2+^ uptake, regulating mitochondrial Ca^2+^ levels and preventing Ca^2+^ overload and consequent mitochondrial dysfunction and cell death under pathological conditions (Waldeck-Weiermair et al., 2010; Zhang et al., 2022).

## 4 Mitochondrial Ca^2+^ overload and permeability transition

Mitochondrial Ca^2+^ uptake is driven by the large negative electrochemical potential (∆*μ*_H_ −180 mV) across the IMM that is maintained by the proton-pumping activity of the, ETC., complexes I, III, and IV (Vasington and Murphy, 1962). If the MCU flux was not modulated by its regulatory subunits, the negative IMM potential would drive large amounts of Ca^2+^ inside mitochondria, overwhelming the buffering capacity of inorganic phosphate contained in the mitochondrial matrix. Mitochondrial Ca^2+^ overload and uncontrolled ROS production lead to the opening of a large pore in the IMM denoted as the mitochondrial permeability transition pore (mPTP), which dissipates the proton-motive force and allows equilibration of solutes <1.5 kD, including proapoptotic factors such as cytochrome *c* (Rasola and Bernardi, 2011). The mPTP plays an important role in regulating cell death and mitophagy under physiological conditions (Bonora et al., 2022), and transient mPTP opening may be necessary to fine-tune mitochondrial Ca^2+^ levels, functioning like a pressure-release valve to prevent Ca^2+^ overload (Lu et al., 2016). Furthermore, mPTP opening is also a central mediator of cardiac myocyte death during ischemia-reperfusion injury (Morciano et al., 2017). Accordingly, MCU-knockout mice exhibit decreased infarct size and improved function after myocardial infarction, as defective Ca^2+^ uptake reduces cardiac myocyte death triggered by mitochondrial permeability transition (Luongo et al., 2015).

A major limitation of inhibiting the mPTP is that the molecular identity of the proteins that compose the pore is largely unknown. Multiple models were developed to explain the molecular architecture of the mPTP, and several proteins including the voltage-dependent anion channel (VDAC), the adenine nucleotide translocator (ANT), and the F_1_-F_o_ ATP synthase (complex V) were proposed to be mPTP component (for a recent review, see (Bauer and Murphy, 2020)). Cyclosporine A inhibits permeability transition by targeting cyclophilin D, which is an activator of mPTP opening. Genetic deletion of *Ppif* worsens cardiac hypertrophy, fibrosis, and myocardial function in response to pressure overload. In *Ppif*-deficient mice, impaired mPTP opening causes an alteration in Ca^2+^ efflux, increasing mitochondrial Ca^2+^ and consequently activating Ca^2+^-dependent TCA cycle dehydrogenases. As a result, glucose oxidation is favored relative to fatty acids, reducing the metabolic flexibility of the heart during stress (Elrod et al., 2010). These findings suggest that the mPTP contributes to metabolic adaptation during changes in cardiac workload.

## 5 Mechano-energetic uncoupling in heart failure

In the failing heart, altered Ca^2+^ handling is a major contributor to contractile dysfunction and arrhythmias (Luo and Anderson, 2013). A primary defect in HF is the reduced Ca^2+^ load of the SR (Lindner et al., 1998), which results from reduced Ca^2+^ reuptake via SERCA and Ca^2+^ release due to spontaneous RyR2 opening. Consequently, the amplitude and decay velocity of cytosolic Ca^2+^ transients are reduced, and [Ca^2+^]_c_ during diastole is increased, thereby lowering systolic force development and increasing wall tension during diastole (Hasenfuss et al., 1996; O’Rourke et al., 2021). Another central alteration is the increase in [Na^+^]_i_, which results from increased late Na^+^ current (*I*_Na_) (Valdivia et al., 2005), increased Na^+^/proton exchanger (NHE) activity (Baartscheer et al., 2003), and decreased Na^+^/K^+^ ATPase activity (Schwinger et al., 1999).

**FIGURE 2:**
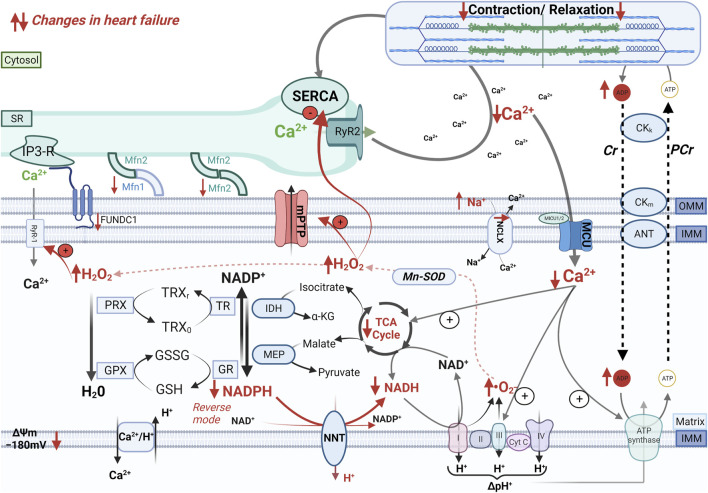
Mechano-energetic uncoupling in the failing heart. In the failing heart, altered mitochondria-SR communication, decreased MCU current, and elevation of cytosolic Na^+^ levels that accelerates Ca^2+^ extrusion via the mitochondrial Na^+^/Ca^2+^ exchanger (NCLX) impair Ca^2+^ accumulation in the mitochondrial matrix during elevations of cardiac workload. The insufficient stimulation of the TCA cycle dehydrogenases causes an oxidation of the mitochondrial pyridine nucleotides, thereby causes bioenergetic mismatch and oxidative stress, which contribute to the progression of heart failure. Furthermore, pathological increase in cardiac afterload can reverse the NNT reaction, which thereby regenerates NADH at the expense of the NADPH pool, thus further draining reducing equivalents from mitochondrial antioxidant systems.

Alterations in cytosolic Na^+^ and Ca^2+^ handling and the resulting changes in mitochondrial Ca^2+^ signals have profound consequences on mitochondrial oxidative metabolism and antioxidant defense. In HF, reduced SR Ca^2+^ load and disruption of the tubular SR network (He et al., 2001) decrease Ca^2+^ concentration in the mitochondria-SR contact sites, thereby impairing mitochondrial Ca^2+^ uptake. In addition, mitoplasts (i.e., mitochondria stripped of the outer membrane) isolated from patients with HF exhibit decreased MCU activity compared with nonfailing controls (Michels et al., 2009), which might be explained by an increased expression of the regulatory subunits MICU1 and MICU2 (Paillard et al., 2022). Furthermore, elevated [Na^+^]_i_ accelerates Ca^2+^ efflux from the mitochondrial matrix via the NCLX (Maack et al., 2006). Although elevated [Na^+^]_i_ also favors Ca^2+^ influx in the cytosol via the reverse mode of the NCX, this is far less efficient in inducing mitochondrial Ca^2+^ uptake compared with SR Ca^2+^ release due to the slower NCX kinetics and the more remote location of the NCX flux compared with the RyR2 flux (Kohlhaas and Maack, 2010). Altogether, derangements in cellular Ca^2+^ and Na^+^ handling contribute to hamper mitochondrial Ca^2+^ signals, thereby hindering the Ca^2+^-mediated stimulation of oxidative metabolism that is required to adapt the production of reducing equivalents for the ETC to the ATP demand of cardiac myocytes (Figure 2).

At the same time, defective stimulation of the TCA cycle by mitochondrial Ca^2+^ leads to an inadequate supply of reducing equivalents required to maintain matrix NAD(P)H redox potential. In a guinea pig model of HF induced by combined chronic β-adrenergic stimulation and pressure overload, pharmacological inhibition of the NCLX could counteract the depletion of mitochondrial Ca^2+^ induced by elevated [Na^+^]_i_, thereby restoring NAD(P)H redox state and reducing ROS emission from mitochondria (Kohlhaas et al., 2010). This intervention ameliorated cardiac remodeling, improved cardiac function and decreased the risk of ventricular arrhythmias (Liu et al., 2014). In an analogous guinea pig model of HF, moderate overexpression of MCU by viral gene transfer reduced oxidative stress induced by increased cardiac workload, improved systolic function and decreased the number of premature ventricular complexes (Liu et al., 2021).

The mechanisms linking restoration of mitochondrial Ca^2+^ levels by NCLX inhibition or MCU overexpression and protection from ventricular arrhythmias and sudden cardiac death in this model are not completely resolved. One hypothesis is that the antiarrhythmic effect of correcting mitochondrial Ca^2+^ levels results from decreased ROS-mediated oxidation of RyR2, which decreases spontaneous SR Ca^2+^ release events that cause delayed afterdepolarizations, a known trigger of arrhythmias (Zima and Blatter, 2006). In mice, SR Ca^2+^ leaks feed a vicious cycle whereby mitochondrial Ca^2+^ mishandling leads to mitochondrial dysfunction, ROS emission, and RyR2 oxidation, further exacerbating proarrhythmic SR Ca^2+^ leaks (Santulli et al., 2015; Hamilton et al., 2020). One additional mechanism might be represented by ROS-induced activation of Ca^2+^/calmodulin-dependent protein kinase II (CaMKII), which prolongs action potential duration by phosphorylating Na^+^ channels (Erickson et al., 2008; Wagner et al., 2011). Therefore, oxidation of mitochondrial pyridine nucleotides and mitochondrial ROS production create both a trigger and a substrate for ventricular arrhythmias, but the underlying mechanisms have not been fully elucidated.

It is important to note that this mechanistic framework is supported by studies in guinea pigs exposed to combined chronic β-adrenergic stimulation and pressure overload, which might explain the apparently conflicting results obtained in smaller rodents subjected to myocardial infarction. In the latter model, it has been reported that mitochondrial Ca^2+^ levels are increased, rather than reduced (Santulli et al., 2015), and abrogating Ca^2+^ efflux by inhibiting the mPTP or the NCLX has beneficial, rather than detrimental effects (Elrod et al., 2010; Boyman et al., 2021). Importantly, guinea pigs more closely recapitulate human excitation-contraction coupling, whereas mice exhibit substantial differences including the higher [Na^+^]_i_ (9–14 mmol/L vs. 4–8 mmol/L) and the smaller contractile reserve compared to humans that might partly explain the seemingly contradictory conclusions of these studies.

Pathological elevations of cardiac workload such as those induced by pressure overload also deplete mitochondrial antioxidative capacity and cause oxidative stress by reversing the reaction catalyzed by the mitochondrial nicotinamide nucleotide transhydrogenase (NNT) (Nickel et al., 2015). Under physiological conditions, the NNT transfers electrons from NADH to regenerate NADPH, thus bolstering mitochondrial antioxidative capacity (Figure 2). Pressure overload reverses the NNT reaction, thereby consuming NADPH to regenerate NADH required to sustain the ECT activity. The ensuing ROS production is a major driver of maladaptive remodeling after transverse aortic constriction in mice (Nickel et al., 2015). The yin/yang role of the NNT in the modulation of mitochondrial ROS emerged from the observation that C57BL/6J mice, an inbred mouse strain carrying a loss-of-function mutation of the *Nnt* gene, are protected from oxidative stress and heart failure induced by pressure overload (Nickel et al., 2015). Therefore, the use of this mouse strain in cardiovascular and/or metabolic research should be avoided.

Altogether, these studies indicate that alterations in cardiac myocyte Na^+^ and Ca^2+^ handling as seen in HF hinder cardiac metabolic adaptation and exacerbate mitochondrial ROS production by preventing Ca^2+^ accumulation in the mitochondrial matrix. In rodent models of HF, counteracting these changes with pharmacologic interventions or genetic manipulation had beneficial effects on cardiac remodeling and arrhythmias.

## 6 Mitochondrial Ca^2+^ handling as a therapeutic target in heart failure

Despite the large body of evidence indicating that interventions aimed at ameliorating mitochondrial Ca^2+^ handling in cardiac myocytes have beneficial effects in cardiac disease, studies supporting this concept in humans remain limited. In the context of I/R injury, mitochondrial Ca^2+^ overload triggers mPTP opening, one central driver of cardiac myocyte loss. Accordingly, studies in rodent models of I/R injury demonstrated that genetic inhibition of the MCU reduced cardiac injury by inhibiting mPTP opening (Kwong et al., 2015; Wu et al., 2020). However, mPTP inhibition by cyclosporine infusion immediately prior to reperfusion did not improve cardiovascular outcomes nor attenuated maladaptive remodeling in patients with myocardial infarction in a phase 3 randomized controlled trial (Cung et al., 2015). One key translational challenge in this context is that mitochondrial Ca^2+^ overload ensues during ischemia, and mPTP opens as soon as the intracellular pH is restored upon reperfusion (Hartmann and Decking, 1999). Therefore, these detrimental processes are already at play when the patient arrives to the catheterization laboratory for percutaneous reperfusion. Viceversa, the therapeutic goal in chronic HF is to restore physiological levels of mitochondrial Ca^2+^, which can be achieved with two different approaches: boosting mitochondrial Ca^2+^ uptake with pharmacological agents or preventing mitochondrial Ca^2+^ efflux either by inhibiting the NCLX or by decreasing [Na^+^]_i_. Pharmacological agents that were tested in human HF an could potentially improve mitochondrial Ca^2+^ handling are discussed below.

### 6.1 Ranolazine

An enhanced late Na^+^ inward current (*I*_Na_) is one important contributor to high [Na^+^]_i_ in HF (Wagner et al., 2006). The selective *I*_Na_ inhibitor ranolazine is approved for symptomatic treatment of stable angina (Silva et al., 2016). In animal models of HF, ranolazine effectively lowered [Na^+^]_i_, improved diastolic function, prevented arrhythmias, and pulmonary hypertension (Uran, 2021). On these grounds, ranolazine was tested in patients with HF with preserved ejection fraction, but did not affect ventricular relaxation in the RALI-DHF trial (Ranolazine in Diastolic HF) (Maier et al., 2013).

### 6.2 Cariproride

Increased activity of the NHE also contributes to the elevation in [Na^+^]_i_ seen in failing cardiac myocytes (Baartscheer et al., 2003). Furthermore, abnormal activation of NHE can increase intracellular pH, thereby enhancing myofilament Ca^2+^ sensitivity and hindering myocardial relaxation, and activate Ca^2+/^calmodulin-dependent protein kinase II (CaMKII), which can further aggravate Ca^2+^ and Na^+^ mishandling in cardiac myocytes and contribute to oxidative stress (Deschaine et al., 2022). The NHE inhibitor cariproride had beneficial effects in rodent models of HF (Baartscheer et al., 2005; Baartscheer et al., 2008), but increased thromboembolic risk in clinical trials (Karmazyn, 2013).

### 6.3 Potential effects of SGLT2i on intracellular Na^+^

Sodium-glucose cotransporter 2 inhibitors (SGLT2i) have demonstrated cardiovascular benefit in patients with acute or chronic HF, with or without diabetes, and independent of left ventricular ejection fraction (Packer et al., 2021a; Packer et al., 2021b; Kosiborod et al., 2022; Voors et al., 2022). The mechanisms underlying their cardioprotective activity are multifactorial and include beneficial effects on systemic metabolism, renal function, hemodynamics, and most likely, direct cardiac effects. It is still unclear whether SGLT2i lower [Na^+^]_i_ and consequently increase mitochondrial Ca^2+^ levels by inhibiting the NHE expressed on cardiac myocytes. Although this model found experimental confirmation in one study performed in isolated ventricular myocytes from rats and rabbits (Baartscheer et al., 2017) and in human atrial cardiac myocytes (Trum et al., 2020), subsequent studies challenged these results (Chung et al., 2021). Another potential mechanism how SGLT2i could reduce [Na^+^]_i_ is inhibition of late *I*_Na_, which was observed in cardiac myocytes from mouse models of HF (Philippaert et al., 2021). Even if [Na^+^]_i_ was not affected, reduction in late *I*_Na_ might have beneficial effects also by reverting action potential prolongation due to increased activity of this current in HF (Philippaert et al., 2021).

## 7 Conclusion

Alterations in Na^+^ and Ca^2+^ handling contribute to contractile dysfunction and arrhythmias in HF. In spite of our detailed mechanistic understanding of these derangements derived from animal models of HF, therapeutic strategies aimed at ameliorating cardiac myocyte ion handling and restoring mitochondrial Ca^2+^ levels have not provided clinical benefit in HF patients thus far. Further studies are needed to understand whether the cardioprotective effects of SGLT2i are mediated, at least in part, by their beneficial activity on mitochondrial Ca^2+^ and cardiac energetics.
